# Effect of Stone-Wales Defect on Mechanical Properties of Gr/epoxy Nanocomposites

**DOI:** 10.3390/polym11071116

**Published:** 2019-07-01

**Authors:** Maoyuan Li, Peng Chen, Bing Zheng, Tianzhengxiong Deng, Yun Zhang, Yonggui Liao, Huamin Zhou

**Affiliations:** 1 State Key Laboratory of Material Processing and Die & Mold Technology, Huazhong University of Science and Technology, Wuhan 430074, China; 2 Key Laboratory of Material Chemistry for Energy Conversion and Storage, Ministry of Education, School of Chemistry and Chemical Engineering, Huazhong University of Science and Technology, Wuhan 430074, China

**Keywords:** Gr/epoxy nanocomposites, defect, molecular dynamic, mechanical properties

## Abstract

Due to its superior mechanical properties, graphene (Gr) has the potential to achieve high performance polymer-based nanocomposites. Previous studies have proved that defects in the Gr sheets could greatly reduce the mechanical properties of Gr, while the Stone-Wales (SW) defect was found to enhance the interfacial mechanical strength between Gr and epoxy. However, the combined effects of defects on the overall mechanical properties of Gr/epoxy nanocomposites have not been well understood. In this paper, the effect of the SW defect on the mechanical properties of Gr/epoxy nanocomposites was systematically investigated by using molecular dynamic simulations. The simulation results showed that the SW defect would degrade the mechanical properties of nanocomposites, including the Young’s modulus and in-plane shear modulus. Surprisingly, the transverse shear modulus could be remarkably enhanced with the existence of SW. The reinforcing mechanisms were mainly due to two aspects: (1) the SW defect could increase the surface roughness of the Gr, preventing the slippage between Gr and epoxy during the transverse shea; and (2) the nanocomposite with defective Gr enables a higher interaction energy than that with perfect graphene. Additionally, the effects of temperature, the dispersion and volume fraction of Gr were also investigated.

## 1. Introduction

Epoxies with cross-linked network structure exhibit excellent mechanical properties, good chemical stability and durability, which makes them widely used in electronic packaging and the automotive and aerospace fields [1,2,3,4]. However, the high cross-linked structure also makes the epoxy have low toughness and poor impact and crack resistance, which limits its application in the high-end fields as a structural material [5,6]. The incorporation of nanofillers was the most common and effective way to improve the mechanical properties of epoxy-based composites [7,8]. Among various fillers, owing to its extraordinary mechanical properties (Young’s modulus is ~1.0 TPa, tensile strength is ~123.5 GPa [9]) and high specific surface area (2630 m^2^/g) [10], graphene (Gr) [11] is regarded as ideal for enhancing polymer-matrix nanocomposites [8,12,13,14].

Numerous experimental works have proven that the mechanical properties of epoxy could be improved with the incorporation of Gr, including Young’s modulus [15], tensile strength, fracture toughness [16] and glass transition temperature [17]. For example, Rafiee et al. [15] found that Gr could significantly improve the Young’s modulus (~31%), tensile strength (~40%) and fracture toughness (~53%) of epoxy at a low addition of 0.1 wt% Gr. Meanwhile, Gr showed a better enhancement effect compared to carbon nanotube, which was attributed to the higher aspect ratio and stronger bonding/interlocking interaction with the epoxy matrix. Previous experimental and simulations results [15,17,18] also indicated that the mechanical properties of Gr/epoxy nanocomposites are mainly determined by the volume fraction, dispersion state and the interfacial mechanical properties between Gr and epoxy. For instance, Tang et al. [17] found that, compared with poor dispersion, well-dispersed Gr could increase the glass transition temperature (11 °C) and tensile strength more effectively, and the fracture toughness increased by ~52% at 0.2 wt % Gr.

Modification of the interfacial properties between Gr and epoxy was also a useful method for improving the mechanical properties of composites. Wan et al. [19] have successfully grafted the diglycidyl ether of bisphenol-A (DGEBA) molecular chain onto the surface of Gr, which effectively improved the dispersibility and the compatibility with epoxy resin. Compared with epoxy resin, the Young’s modulus and strength of the composite increased by ~13% and 75%, respectively. The theoretical analysis and simulations also verified the experimental results and shed light on the enhancement mechanism from the perspectives of microstructure and energy variation. Using the molecular dynamic (MD) simulations, Skountzos et al. [20] indicated that the interaction energy could be significantly enhanced by the chemical modification of Gr and the surface adsorption on the polymer was improved, leading to the improved mechanical properties of composites. 

The above research has improved our understanding of the mechanical properties of Gr/epoxy nanocomposites but so far, there are few comprehensive studies on the mechanical properties of epoxy-based composites containing defective Gr, and many efforts have been made to prepare defect-free Gr for achieving high mechanical performance of composites. Nevertheless, the defects [21,22] in the Gr, such as vacancy, Stone-Wales (SW) and add-atoms defects are unavoidable during the fabrication process. According to our recent work [23,24] and some previous literature [25,26,27], the mechanical properties of Gr would decrease significantly with the existence of defects even at a rather low defect concentration. Moreover, our recent simulation results [28] also showed that vacancy defects would degrade the interfacial mechanical strength between Gr and epoxy, while it could be improved by the SW defect.

However, the influences of SW defects on the overall mechanical properties of Gr/epoxy composites have not well been understood. Since the defect is inevitable, it is significant to fully understand: (a) the combined effects of the degradation in the mechanical properties of Gr and enhancement in the interfacial mechanical strength caused by SW defects; and (b) the underlying enhancement or degradation mechanism of SW defects on the Gr/epoxy nanocomposites.

In this study, we have conducted a series of molecular dynamic simulations to provide a direct insight into the effect of defects on the mechanical properties of Gr/epoxy nanocomposites. The SW defect was randomly dispersed in Gr sheets. The surface roughness of Gr and the interaction energy between Gr and epoxy were analyzed and compared to clarify the mechanisms of the mechanical properties of nanocomposites. Additionally, the effects of the temperature, dispersion and volume fraction of Gr were also investigated.

## 2. Modeling and Simulation

### 2.1. Molecular Model

**Figure 1:**
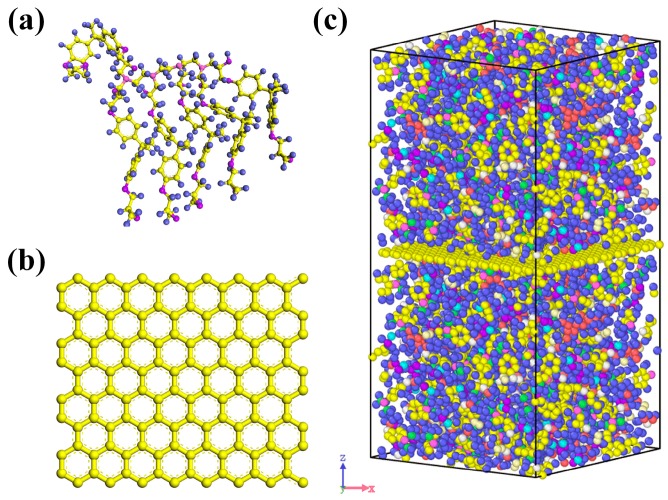
The molecular model of (**a**) representative molecular, (**b**) pristine Gr and (**c**) Gr/epoxy nanocomposite. (blue balls are hydrogen atoms, purple balls are oxygen atoms, lilac balls are nitrogen atoms, yellow balls are carbon atoms in epoxy, respectively).

Molecular dynamic (MD) simulations were used to explore the mechanical properties of Gr/epoxy nanocomposites and molecular models were first constructed using commercial software—Material Studio (Accelrys). The representative molecular method proposed by Yu et al. [29] was employed to construct the initial epoxy model and this method has been successfully used to obtain the mechanical [30] and thermal properties [31] of the cross-linked epoxy, of which the results were quite comparable with experimental results [32]. The epoxy model was composed of bisphenol A diglycidyl ether (DGEBA) as the resin matrix and triethylenetetramine (TETA) [33] as the curing agent, as shown in Figure 1a. The nanocomposite model consisted of 10,200 atoms, with a dimension of 3.8 nm × 3.8 nm × 7.8 nm, while the *x* and *y* directions were the armchair and zigzag directions, respectively. Since the Gr sheet scale for the Gr/epoxy composite system via molecular dynamic simulations was limited and much smaller than those observed by AFM/SEM/TEM (~um), the periodic boundary conditions could prevent the surface effect of the finite-sized unit cell structure. The nanocomposites model consisted of Gr sheets with or without defects, sandwiched between two epoxy blocks, as shown in Figure 1c. The molecular models can reflect the case that the composites were fabricated via layer by layer assembly [34], the Gr fully exfoliated in the polymer matrix can be assumed to have large dimensions as well as planar orientation. Meanwhile, such molecular models have been widely used to investigate the mechanical properties of Gr based nanocomposites in previous studies [30,35,36].

The volume fraction *V_gr_* could be transformed by the mass fraction *w_gr_*:

(1) $$Vgr=\frac{w_{gr}}{w_{gr}+\left(\rho_{gr}/\rho_{m}\right)(1-w_{gr})}$$

where *ρ_gr_*, *ρ_m_* represents the density of Gr and epoxy, respectively. The *ρ_m_* = ~1.1 g/cc [31], *ρ_gr_* = ~4.12 g/cc [36]. According to equation (1), the weight fraction of Gr *w_gr_* was 9.0%, its volume fraction *V_gr_* was ~2.7%.

### 2.2. Force Field

An ab initio force field, the polymer consistent force field (PCFF) [37] based on CFF91 with additional parameters, was used to simulate inter-atomic interaction for the epoxy molecular as it has been successfully employed to investigate the mechanical properties of various polymers, for example, polyethylene, epoxy resin [38]. The function form of the PCFF potential consisted of bonded and non-bonded energy, the former included the energy for bond stretching, angle bending, torsion, inversion, and the cross term of these functions. The interactions for the carbon atoms in Gr was described by Tersoff potentials [39,40]. With no chemical bonds between Gr and epoxy, the interactions between them were described by 12-6 Lennard-Jones (LJ) potentials, which can also be called a van der Waals interaction between Gr and the epoxy matrix. The 12-6 LJ potential was described as *E_ij_* = 4*ε_ij_* [2(*δ*/*r_ij_*)^12^−(*δ*/*r_ij_*)^6^], where *r_ij_* is the distance between atom *i* and *j*, *ε* and *δ* are the energy and distance constants, respectively. The LJ potential parameter between different types of atoms were calculated by using the Lorentz-Berthlot rules (i.e., $\varepsilon_{ij}=\sqrt{\varepsilon_{i}\varepsilon_{j}}$; $\delta_{ij}=\left(\delta_{i}+\delta_{j}\right)/2$). The LJ potential parameters for different atoms are listed in Table 1. A potential cut-off of 1.0 nm was used for the calculation of nonbonded interactions. The Large-scale Atomic/Molecular Massively Parallel Simulator (LAMMPS) [41] was used to conduct all the molecular dynamic simulations and the velocity-Verlet method was used to integrate the equation of motion.

### 2.3. Calculation Method

**Figure 2:**
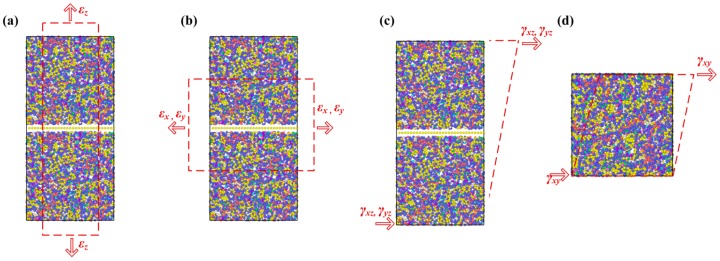
Deformation of Gr/epoxy nanocomposite: (**a**) longitudinal tension, (**b**) transverse tension, (**c**) longitudinal shear and (**d**) in-plane shear.

After the initial model was established, the mechanical properties of Gr/epoxy nanocomposites were investigated by uniaxial tension and shear deformation using MD simulations, as shown in Figure 2. Before deformation, the initial structure was relaxed to reach an equilibrium state; the relaxation involved three different steps. At the beginning, an energy minimization was performed using the conjugate gradient algorithm. To eliminate the internal stress, an annealing process was applied, that is, the system was relaxed in an NVT (i.e., constant number of atoms, volume and temperature) ensemble, during which the temperature was increased from 100 K to 1000 K at rate of 9 K/ps, and then kept in 1000 K for 200 ps. Then it was cooled down to the desired temperature of 100 K at the same rate. Afterwards, the system was fully relaxed in an isothermal-isobaric NPT ensemble (i.e., constant number of atoms, pressure and temperature) at 100 K and 1 atm for 500 ps. Followed by the equilibration, a constant uniaxial strain was applied along along *x*, *y* or *z* directions at a strain rate of 0.001 ps^−1^. The shear deformation was performed along *xy*, *xz* or *yz* directions at a shear rate of 0.001 ps^−1^. During the deformation process, the system was relaxed in NVT for another 10 ps at 1 ps intervals (i.e., the strain was 0.001). The atomic stress can be calculated by the virial theorem:

(2) $$\sigma_{i}^{\alpha \beta }=\frac{1}{\Omega_{i}}\left\{-m_{i}v_{i}^{\alpha }v_{i}^{\beta }+\frac{1}{2}\sum_{j\neq i}F_{ij}^{\alpha }r_{ij}^{\beta }\right\}$$

where Ω*_i_*, *m_i_* and *v_i_* represent the volume, mass and velocity of atom *i*, respectively; *F_ij_* and *r_ij_* are the force and distance between atom *i* and *j*, respectively. The superscript *α*, *β* denote the Cartesian coordinate components. By averaging the stresses of all the atoms in the system, the mechanical properties of nanocomposites, including Young’s modulus and shear modulus could be obtained from the elastic stage (strain < 0.02) in the stress–strain curves.

Although the mechanical properties of Gr exhibit a typical anisotropic behavior [23], the transverse Young’s and shear modulus of composites is close [42]. Therefore, Young’s modulus and shear modulus along the transverse direction can be determined by *x*, *y*, *xz* and *yz* directions, respectively:

(3) $$E_{T}=\left(E_{x}+E_{y}\right)/2$$

(4) $$G_{L}=\left(G_{yz}+G_{xz}\right)/2$$

The longitudinal Young’s modulus *E_z_* and the in-plane shear modulus *G_xy_* can be determined by the deformation process in the *z* and *xy* directions, respectively. Three independent simulations were carried out under different initial conditions, and the final value was the average of all calculated values.

## 3. Results and Discussions

### 3.1. Validation of Models

To validate the molecular models and the force field, the mechanical properties and density of pure amorphous cross-linked epoxy were calculated. Pure cross-linked epoxy with a block size of 3.6 nm × 3.6 nm × 11.1 nm and 14,490 atoms were used. The final value was the average of three independent simulations results and error bars were determined by the standard deviation. After the calculation, the density of pure epoxy was about 1.08 g/cm^3^, which is close to the experimental and simulation value [31], i.e., ~1.1 g/cm^3^.

**Figure 3:**
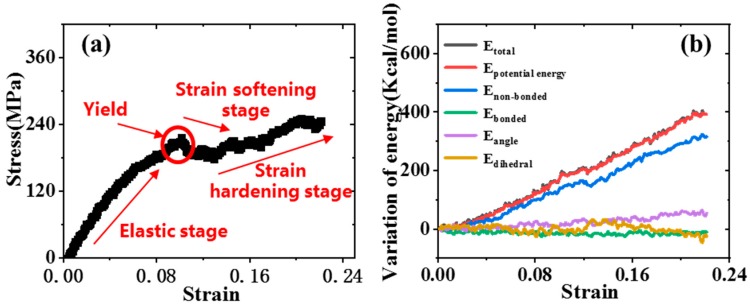
(**a**) The stress-strain curve of pure cross-linked epoxy under uniaxial tension, (**b**) the variation of total energy during tension.

Figure 3 shows the stress-strain curve and the corresponding energy variation during the deformation. To eliminate the statistical uncertainty arising from the thermal fluctuation, the simulations were performed at a relatively low temperature of 100 K unless otherwise stated. The yield point in this case was defined as the stress level at which the epoxy ceased to behave elastically and the stress divided by the strain was no longer constant. In Figure 3a, the stress-strain curve can be divided into four stages during the whole process, namely elastic, yield, strain softening and strain hardening stages. In the initial stage, the stress increased linearly with the strain, indicating that the material was in the elastic deformation stage. Upon reaching the yield point, the stress then showed a decrease to a local minimum, suggesting material softening. As the strain continued to increase, the molecular chains began to orient along the stretching direction, leading to the strain hardening. As shown in Figure 3b, the variation of also showed that there was no obvious temperature variation during the stretching and the variation of the total energy was mainly attributed to the potential energy. The total potential energy of composites consists of bonded and non-bonded energy. The bonded energy included the bond stretching term (bond energy, *E*_bonded_), angle bending term (bending energy, *E*_angle_), and torsion angle term (dihedral energy, *E*_dihedral_), while the non-bonded energy was mainly determined by the vdW energy. In the elastic stage, *E*_bonded_, *E*_angle_ and *E*_dihedral_ changed moderately and the increase of system energy mainly came from the increase of non-bonded energy (*E*_non-bonded_), indicating that the van der Waals force dominated the elastic stage. As the strain increased, the corresponding *E*_bonded_, *E*_angle_ and *E*_dihedral_ began to change due to the deformation of the molecular bonds, such as stretching and bending. The above behavior was consistent with the trend of previous experiments [43,44] and simulations [29,45]. In addition, the Young’s and shear modulus were obtained according to the initial elastic stage of tensile/shear stress-strain curves. The calculated results and the relevant values obtained by previous experiments or simulations are displayed in Table 2. It can be seen in Table 2 that the calculated results agreed with experimental or simulation results, which verified the accuracy of the calculation model and force field used in the present work.

### 3.2. Mechanical Properties of Gr/epoxy Nanocomposites

**Figure 4:**
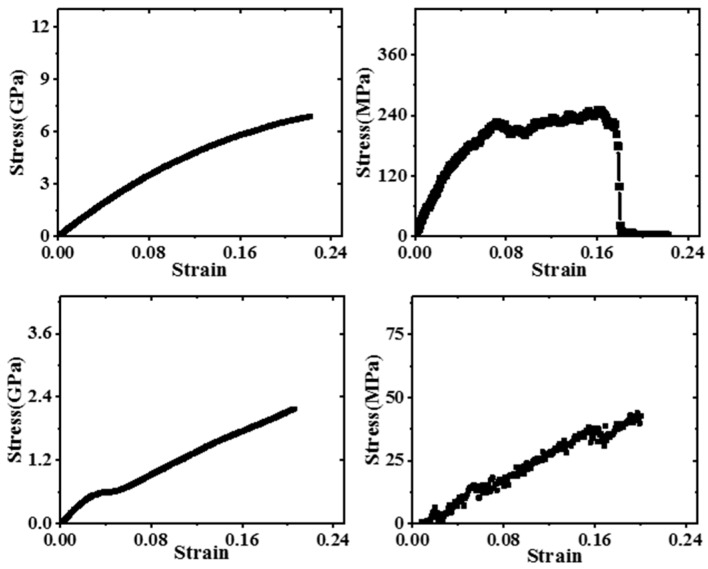
The stress-strain of pristine Gr/epoxy nanocomposite under (**a**) transverse tension, (**b**) longitudinal tension, (**c**) in-plane shear and (**d**) transverse shear.

The mechanical properties of Gr/epoxy nanocomposite without defects were studied. Figure 4 shows the stress-strain curves for different deformations, including transverse (*x*, *y*) and longitudinal (*z*) tension, in-plane (*xy*) and transverse (*xz*, *yz*) shear. This indicates that the stress level of the nanocomposites was much higher than that of the pure epoxy resin in the transverse tension and in-plane shear. This was mainly due to the extremely high Young’s (~936 GPa [23]) and shear modulus (~280 GPa [48]) of Gr. Meanwhile, the periodic boundary condition was applied in the in-plane direction, which was equivalent to the Gr sheets with extremely large dimensions completely exfoliated embedded in the polymer matrix, so the transverse tension and in-plane shear behavior of composites were dominated by the mechanical properties of Gr. In the longitudinal tension and transverse shear, the nanocomposite exhibited similar deformation behavior to the epoxy, and the stress level was close, which was mainly due to the weak van der Waals interaction between the Gr and the epoxy matrix.

In order to quantify the enhancement of Gr, the corresponding Young’s modulus and shear modulus were calculated, as shown in Table 3. The results showed that the mechanical properties of epoxy can be significantly enhanced by the incorporation of Gr. For example, the Young’s modulus (46.12 ± 2.21 GPa) in the transverse direction and in-plane shear modulus (21.04 ± 1.36 GPa) of composites were much larger than 3.45 and 1.35 GPa of the pure epoxy. The longitudinal Young’s modulus (4.62 Gpa ± 0.45) was also improved by the Gr, which was ~34% higher than that of pure epoxy resin. However, the transverse shear modulus (0.40 ± 0.05 GPa) decreased significantly compared to pure epoxy resin. This was mainly because the interfacial shear strength between Gr and epoxy was low and the cohesive strength was large. According to our recent work [28], the interfacial shear strength (~111.10 MPa) was much smaller than the interfacial cohesive strength (~962.77 MPa), thus the slippage was more likely to occur in transverse shear. The above results were also consistent with those of Lin et al. [36] regarding Gr/PMMA composites and Wang et al. [49] regarding the Gr/epoxy composites.

Many micromechanical models have been developed to evaluate the effective material properties of two-phase composites, of which the rule of mixture was commonly used. According to the rule of mixture [50], the effective Young’s modulus and shear modulus can be obtained by:

(5) $$E_{com}=\eta_{1}E_{gr}V_{gr}+E_{ep}V_{ep}$$

(6) $$\frac{\eta_{2}}{G_{com}}=\frac{V_{gr}}{G_{gr}}+\frac{V_{ep}}{G_{ep}}$$

where the subscripts *com*, *gr*, *ep* represent the composite materials, Gr and epoxy, respectively. The *V* represents the volume fraction and *V_gr_* + *V_ep_* = 1. η is the scale-dependent parameters related to the shape and size of the material. When η = 1, the formula (5)–(6) reduces to the classical mixing law of the two-phase composite material. Due to its unique two-dimension structure, the longitudinal tensile modulus and transverse shear modulus of Gr cannot be determined; thus, the transverse Young’s modulus and in-plane shear modulus were discussed here. By using *E_gr_* = 936 GPa, *G_gr_* = 280 GPa, *E_ep_* = 3.45 GPa, *G_ep_* = 1.35 GPa, the parameter η can be obtained by fitting the rule of mixture. As shown in Table 3, η_1_ = 1.69 > 1, η_2_ = 15.16 > 1, compared with the classical rule of mixture (η = 1), the transverse Young’s modulus and in-plane shear modulus of the nanocomposite exhibited a stiffness-enhancement effect. Moreover, by introducing a larger parameter η, the results obtained by the rule of mixture can be in consistent with the MD simulations [51].

### 3.3. Effect of Dispersion and Temperature

**Figure 5:**
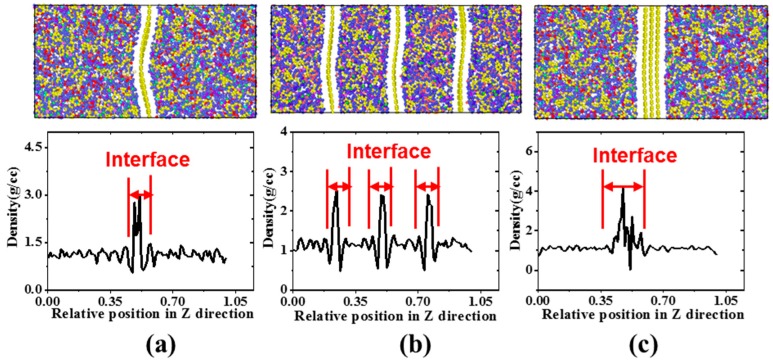
The density of Gr/epoxy nanocomposite along the z direction for (**a**) single Gr, (**b**) dispersed Gr and (**c**) aggregated Gr.

Previous experiments [15,17] have shown that the dispersion and temperature have an important influence on the mechanical properties of Gr based composites. Meanwhile, the well dispersion of Gr plays an important role in achieving high mechanical or thermal performance for nanocomposites. Therefore, we investigated the mechanical properties of composites with three types of Gr/epoxy system at different temperatures (1, 100, 300 K). Figure 5 showed the density distribution and the molecular models for three types of Gr/epoxy nanocomposites along the *z*-direction. Due to the thermal fluctuations and the interactions with epoxy matrix, the Gr sheets were no longer flat after equilibrium, which were also in consistent with previous simulation [52] and experimental results [15]. The density of composites remained a constant value in the pure epoxy region (~1.1 g/cc), the peak appeared at the location of Gr, and the density of the epoxy near the Gr also increased, indicating the interface formed by the van der Waals between Gr and epoxy. As shown in Figure 5, the composites with well-dispersed Gr owned more interfacial regions and more high-density epoxy regions were formed. The interatomic distance between the epoxy layer and Gr sheet was determined by the vdW interactions between the carbon atoms in Gr and the atoms in epoxy. Initially, the distance between epoxy and Gr sheet was maintained ~2 Å. However, it is hard to measure the interatomic distance after equilibrium due to the wrinkle of Gr. According to our recent work [28], the interaction energy between Gr sheets was much stronger than that between Gr and epoxy, leading to a higher interaction shear strength. Previous literature [8,35,53] has also shown that the interfacial regions between Gr and the polymer matrix have a significant effect on the mechanical properties of the composite, for example, Hu et al. [34] embedded the Gr oxide sheets into the polymer matrix by way of layer-by-layer. A dense network structure and interfacial regions were formed by the hydrogen bonding, van der Waals, and so forth, thereby achieving a very high Young’s modulus (~145 GPa).

The Young’s modulus and shear modulus at different temperatures for three types of composites were listed in Table 4. It showed that the tensile modulus and in-plane shear modulus increased with the increase of Gr volume fraction, while the longitudinal shear modulus was lower than that of pure epoxy resin. For example, when the volume fraction of Gr increased from 2.7% to 8.1%, the transverse tensile modulus of the well-dispersed Gr composite increased by ~154% (46.12 to 117.28 GPa) and the in-plane shear modulus increased by ~148% (21.04 to 52.11 GPa), which was mainly due to the dominated mechanical properties by Gr for the transverse and in-plane shear. Meanwhile, the transverse Young’s modulus (133.23 GPa) of the nanocomposite with aggregated Gr was larger than that of the nanocomposite with dispersed Gr (117.28 GPa). The main reason was that the interaction force between Gr was greater than that between Gr and epoxy, the aggregated Gr was more likely to maintain flatness during the deformations (Figure 5c), leading to a larger tensile modulus. However, in the longitudinal stretching, the nanocomposite with well-dispersed Gr owned a larger modulus (6.61 GPa) than that with aggregated Gr (5.56 GPa), which was mainly due to the more interfacial regions formed by the well-dispersed Gr and epoxy. Moreover, the nanocomposite with the single-layer Gr possessed a larger longitudinal shear modulus (~0.47 GPa) than that with aggregated Gr (~0.43 GPa), which also was consistent with our recent results that the interfacial shear strength would be reduced by the agglomeration of Gr [28].

According to Table 4, the mechanical properties, including the tensile modulus and shear modulus in different directions decreased as the temperature increased from 1 to 300 K. Due to the higher thermal vibration and further oscillation of atomic displacement, the longitudinal shear modulus of the nanocomposite system cannot be obtained under 100 and 300 K. Meanwhile, the mechanical properties of nanocomposite became more sensitive to the temperature with the increase of volume fraction. For example, when the temperature increased from 1 to 300 K, the transverse Young’s modulus of the nanocomposite with a volume fraction of 2.7% decreased by ~10.7% (48.81 to 43.57 GPa), while it decreased by ~14.8% (123.43 to 105.15 GPa) for the nanocomposite with a volume fraction of 8.1%. This was mainly due to the temperature-dependent behavior for the mechanical properties of Gr [23].

### 3.4. Effect of Defects

According to our recent works [23,28], the mechanical properties of Gr would be significantly weaken with the introduce of defects, including SV, DV and SW defects, while the existence of SW defect was found to enhance the interfacial mechanical properties of Gr/epoxy. The combined effect of the degradation of the mechanical properties of Gr and enhancement of the interfacial mechanical properties on the overall mechanical properties of nanocomposites was not clear. Therefore, the effect of SW defect was discussed in this section.

**Figure 6:**
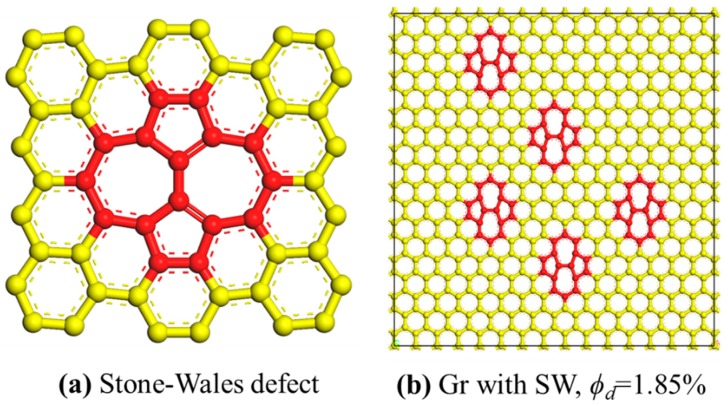
The molecular model of (**a**) Stone-Wales defect; (**b**) Gr sheet with SW, ϕd = 1.85%.

**Figure 7:**
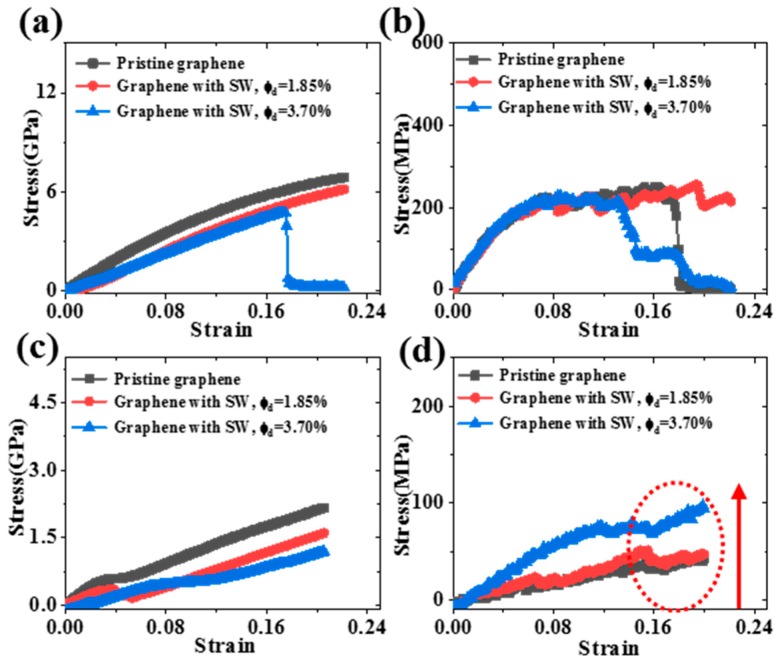
The stress-strain curves of nanocomposite with different concentrations of SW defect under (**a**) transverse tension, (**b**) longitudinal tension, (**c**) in-plane shear and (**d**) transverse shear.

The Figure 6 showed the molecular models of SW defect and the Gr sheet with SW defects. The SW defect was created by rotating one of the C–C bonds by 90°, transforming four hexagons into two pentagons and heptagons (5-7-7-5). The concentration ranged from 0% to 3.70%. The concentration was defined as the number density of defective carbon atoms, and a SW defect was considered for two defective atoms. Meanwhile, the SW defect was randomly distributed on the Gr sheet. Figure 7 was the stress-strain curves of the Gr/epoxy nanocomposite with different SW defect concentrations under different deformation conditions. According to stress-strain curves, the Young’s and shear modulus at different SW defect concentrations were calculated, as shown in Table 5. The results showed that the transverse Young’s modulus and in-plane shear modulus decreased with the increase of SW defect concentration, which were dominated by the mechanical properties of Gr, as shown in Figure 6a. The corresponding stress level and the fracture strength of the nanocomposite decreased significantly. Surprisingly, the longitudinal shear modulus of the composite was significantly enhanced by the existence of SW defect. For example, when the defect concentration was 3.70%, the longitudinal shear modulus increased by ~133% (0.40 to 0.93 GPa). Moreover, the maximum longitudinal shear stress level of nanocomposites containing SW defect was also significantly improved (40 to 80 MPa) compared to that containing Gr without defect.

**Figure 8:**
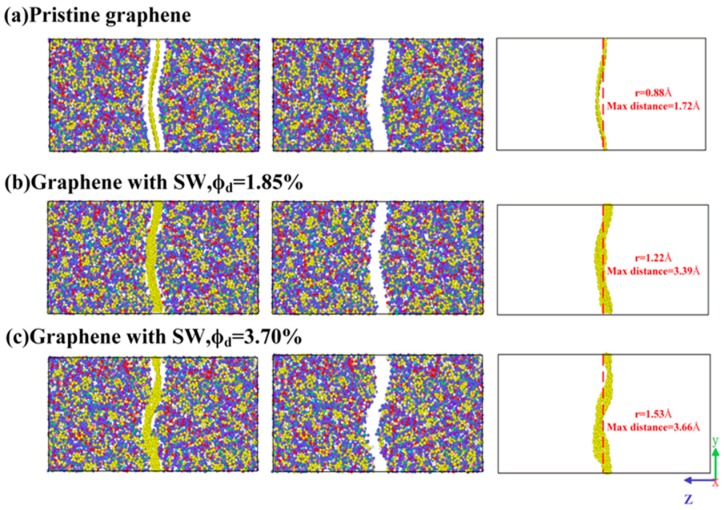
The molecular models of graphene, epoxy and nanocomposite with different concentrations of SW defect.

Next, the enhancement mechanisms of SW defect on longitudinal shear modulus were discussed. Figure 8 showed the fully relaxed molecular model of Gr, epoxy and nanocomposites with different defect concentrations. It clearly indicated that the degree of wrinkle of graphene increased significantly with the increase of SW defect concentration. To quantitatively characterize the degree of wrinkle of graphene, the surface roughness of Gr was defined as the average value of differences between coordinates in the *z* direction of the carbon atoms and the centroid atom. The coordinates of carbon atoms and centroid atom were determined by the status before and after equilibrium, respectively.

(7) $$r=\frac{\sum_{i}\left|z_{i}-z_{center}\right|}{N}$$

**Figure 9:**
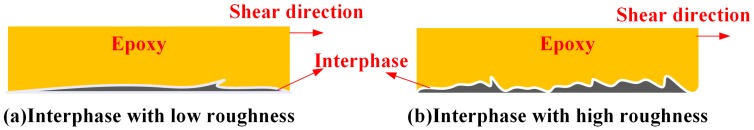
Schematic diagram of shear deformation of nanocomposite along longitudinal direction under different interface roughness.

N is the number of carbon atoms, z_i_ and z center represent the coordinates in the *z* direction of the i-th atom and the centroid atom, respectively. According to Equation (7), the surface roughness of Gr with different SW defect concentrations was 0.88, 1.22 and 1.53 Å, respectively. The degree of Gr wrinkles increased with the increase of SW defect concentration, leading to the increase of surface roughness. Meanwhile, the maximum displacement relative to the centroid atom increased with the existence of SW defect: 1.72 Å < 3.39 Å < 3.66 Å. Figure 9 showed the process of shear deformation along the longitudinal direction of the composites system with different interface roughness. During the longitudinal shear deformation process, the interfacial strength was determined by the slippage between the Gr and epoxy. The increase of the surface roughness can effectively prevent the slippage and increase the shear stress, thereby increasing the longitudinal shear modulus. This was also consistent with our recent results, i.e., SW defects can effectively increase the interfacial shear strength of Gr/epoxy. The previous study [52] also showed that the force (~142 kcal/molÅ) required to pull the wrinkled Gr out of the polymer matrix was much larger than that of the flat graphene (~58 kcal/molÅ).

On the other hand, the interfacial interactions energy plays a key role in determining the overall mechanical properties. The interaction energy between Gr and epoxy *E*_inter_ [54]:

(8) $$E_{\mathrm{int}er}=E_{composite}-\left(E_{graphene}+E_{epoxy}\right)$$

**Figure 10:**
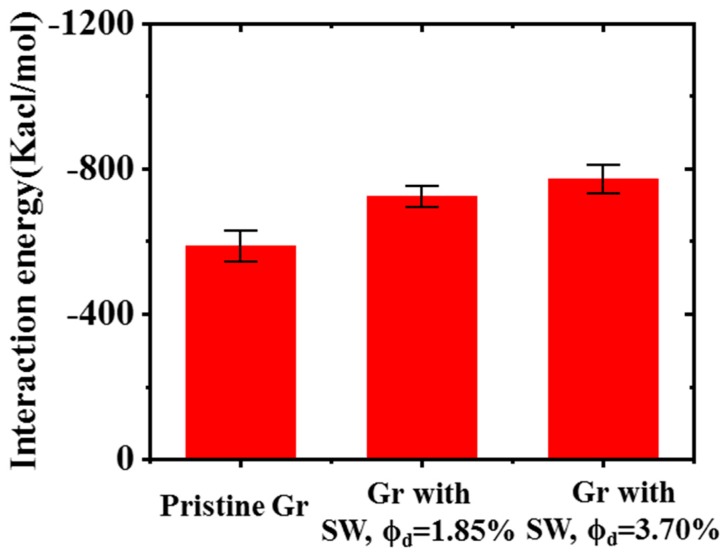
The interaction energy of Gr/epoxy with different concentrations of SW defect.

*E*_composite_, *E*_graphene_, and *E*_epoxy_ represent the energy of composites, Gr, and epoxy, respectively. *E*_graphene_ and *E*_epoxy_ can be calculated separately by removing epoxy or Gr (Figure 8). As shown in Figure 10, the interaction energy increased significantly with the increase of SW defect concentrations. Larger interaction energy can be beneficial to improve the mechanical properties of the composites, especially for the longitudinal shear deformation. Therefore, the improved longitudinal shear modulus of nanocomposites caused by SW defect can be attributed to the above two enhancement mechanisms. This was also consistent with our recent results [28], i.e., SW defects can effectively increase the interfacial shear strength of Gr/epoxy. The introduction of SW defect was found to enhance the cohesive strength and shear strength, with a maximum increase of ~4%. The underlying enhancement mechanism can be explained by the fact that the absorbed energy between the SW defective Gr and epoxy was stronger than that for the pristine Gr. In addition, the existence of SW would increase in π–π attractions at the Gr–epoxy interface, leading to a better interfacial load transfer in Gr–epoxy system. The above results can also provide a certain theoretical explanation for some previous experimental findings. Yang et al. [55] found that the interfacial shear strength (IFSS) of the carbon nanotube/polymer-derived ceramic could be effectively enhanced by ~154% with the existence of defects formed by high-energy carbon ions, while the fracture strength of the composites slightly decreased. In a recent work, Chu et al. [56] also found that the strength of Gr-reinforced copper composite could be enhanced by the surface defect introduced by the plasma treatment, and the interface exhibited better stability under thermal cycling. The intrinsic defects of Gr are unavoidable during synthesis process, and the tailoring of interfacial strength by the defect might provide a new idea for improving the mechanical properties of composites.

## 4. Conclusions

In summary, the mechanical properties of Gr/epoxy nanocomposite were investigated by MD simulations in present study, and the effects of temperature, dispersion and defect were discussed. Due to the temperature-dependent behavior of mechanical properties of Gr, the mechanical properties of composite decreased with the increase of temperature, and the sensitivity to temperature increased with the volume fraction. Since the mechanical properties of Gr were significantly decreased by the defect, the longitudinal Young’s modulus and in-plane shear modulus would be significantly degraded with the existence of SW defect. However, the transverse shear modulus could be surprisingly improved with the increase of SW defect concentration. When the SW concentration increased from 0 to 3.70%, the transverse shear modulus increased by ~133% (0.40 to 0.93 GPa). The enhancement mechanism can be attributed to two aspects: on one hand, the existence of SW defect could increase the wrinkle of Gr, leading to a lager surface roughness, which could effectively prevent the slippage between Gr and epoxy during longitudinal shear deformation. On the other hand, the interaction energy could be significantly improved by the SW defect, improving the mechanical properties of composites.

The above findings could provide a comprehensive understanding in the mechanical properties of Gr/epoxy nanocomposites, especially for the effect of defect. Many efforts have been made to prepare the perfect Gr in achieving high performance of nanocomposites, however, the intrinsic defects were unavailable. The combined effects of degradation in the mechanical properties of Gr and enhancement in the interfacial strength caused by SW defect could provide new possibility to improve the mechanical properties of Gr/epoxy nanocomposites.

## Figures and Tables

**Table 1:** LJ potential parameters for different atoms.

Atom Type	Energy Constant ε (kcal/mol)	Distance Constant σ (Å)
carbon atom in Gr	0.064	4.01
aromatic carbon	0.064	4.01
aliphatic carbon	0.054	4.01
nitrogen atom	0.065	4.07
oxygen atom	0.24	3.535
hydrogen atoms attached to carbon	0.02	2.995
hydrogen atoms attached to oxygen	0.013	1.098

**Table 2:** The calculated mechanical properties of pure cross-linked epoxy and relevant values obtained by previous simulations or experiment.

References	Method	Young’s Modulus (GPa)	Shear Modulus (GPa)
Yu (2009) [29]	MD	3.36	1.22
Alian (2015) [46]	MD	3.2	1.1
Littell (2008) [44]	Experiment	2.9	1.07
Cha (2017) [47]	Experiment	3.35	—
This work	MD	3.45 ± 0.03	1.35 ± 0.15

**Table 3:** The Young’s and shear modulus of nanocomposite by MD and the rule of mixture.

Vgr	MD Results				The Rule of Mixture			
2.7%	E_T_ (GPa)	Ez (GPa)	Gxy (GPa)	G_L_ (GPa)	E_T_ (GPa)	Ƞ_1_	Gxy (GPa)	Ƞ_2_
	46.12 ± 2.21	4.62 ± 0.45	21.04 ± 1.36	0.40 ± 0.05	46.12	1.69	21.04	15.16

**Table 4:** The calculated Young’s and shear modulus of nanocomposite with three different morphology at different temperature.

		1 K (GPa)	100 K (GPa)	300 K (GPa)
Single Gr	E_T_	48.81 ± 1.37	46.12 ± 2.21	43.57 ± 5.72
	E_z_	5.32 ± 0.31	4.62 ± 0.45	3.83 ± 1.21
	G_L_	0.47 ± 0.07	0.40 ± 0.05	—a
	G_xy_	21.25 ± 1.07	21.04 ± 1.36	16.53 ± 2.33
Dispersed Gr	E_T_	123.43 ± 3.45	117.28 ± 4.12	105.15 ± 7.27
	E_z_	7.37 ± 0.48	6.61 ± 0.77	5.98 ± 1.36
	G_L_	0.35 ± 0.12	—	—
	G_xy_	55.91 ± 2.67	52.11 ± 3.11	39.63 ± 5.41
Agglomerated Gr	E_T_	137.13 ± 4.15	133.23 ± 6.48	120.31 ± 7.11
	Ez	6.02 ± 0.74	5.56 ± 0.91	3.04 ± 1.21
	G_L_	0.43 ± 0.11	—	—
	G_xy_	51.50 ± 3.86	47.46 ± 2.54	43.93 ± 3.14

^a^—Indicates that the corresponding result can‘t be calculated under the current condition.

**Table 5:** The Young’s and shear modulus of nanocomposite with different concentrations of SW.

	E_T_ (GPa)	E_z_ (GPa)	G_L_ (GPa)	G_xy_ (GPa)
Pristine graphene	46.12 ± 2.21	4.62 ± 0.45	0.40 ± 0.05	21.04 ± 1.36
Graphene with SW, ϕd = 1.85%	21.4 ± 3.17	1.88 ± 0.32	0.77 ± 0.07	11.63 ± 1.58
Graphene with SW, ϕd = 3.70%	24.86 ± 4.65	1.92 ± 0.76	0.93 ± 0.12	8.82 ± 2.14
